# Supplementary material for: Necroptosis enhances ‘don’t eat me’ signal and induces macrophage extracellular traps to promote pancreatic cancer liver metastasis
Source: Nat Commun. 2024 Jul 18;15:6043. doi: 10.1038/s41467-024-50450-6 (PMC11258255; doi:10.1038/s41467-024-50450-6)
Supplement: Supplementary file 1 — Supplementray Information [file 41467_2024_50450_MOESM1_ESM.pdf]

Necroptosis enhances “don’t eat me” signal and induces macrophage extracellular traps to promote pancreatic cancer liver metastasis

Cheng-Yu Liao, Ge Li, Feng-Ping Kang, Zu-Wei Wang, Yi-Feng Tian, Shi Chen et al.

Supplementary Information (SI)

1. **Supplemental figures**

   Supplementary Fig. 1 RNA-seq analysis of T1M0 and T1M1 PDAC and the potential reasons for MLKL elevation.

   Supplementary Fig. 2 The association between survival and MLKL expression in PDAC.

   Supplementary Fig. 3 The expression and activation mode of necroptosis.

   Supplementary Fig. 4 The effects of MLKL knockout and mouse models.

   Supplementary Fig. 5 The macrophage annotation of different clusters according to scRNA-seq.

   Supplementary Fig. 6 MLKL-driven necroptosis recruits and activates macrophages.

   Supplementary Fig. 7 Macrophage phagocytosis events and the upregulation of CD47 through IL6 were measured.

   Supplementary Fig.8 MLKL-driven necroptosis trigger METs formation through Supernatant.

   Supplementary Fig. 9 MLKL-driven necroptosis releases CXCL8 to trigger METs formation.

   Supplementary Fig. 10 CXCL8 promotes EMT and upregulates ICAM1 in tumour cells.

   Supplementary Fig. 11 METs create an ECM-degrading niche.

   Supplementary Fig. 12 Construction and validation of MLKL-overexpressing KDOs.

   Supplementary Fig. 13 Graphical Abstract of proposed mechanism through which necroptosis promotes liver metastasis in PDAC.

2. **Supplemental tables**

   Supplementary Table 1. Detailed characteristics of the enrolled PDAC patients who underwent magnetic resonance imaging.

   Supplementary Table 2. Baseline characteristics of MLKL in clinical PDAC samples.

   Supplementary Table 3. The sequences of primers.

   Supplementary Table 4. The catalogue numbers and dilutions and of antibodies.

Supplemental figures

Supplementary Fig. 1 RNA-seq analysis of T1M0 and T1M1 PDAC and the potential reasons for MLKL elevation.

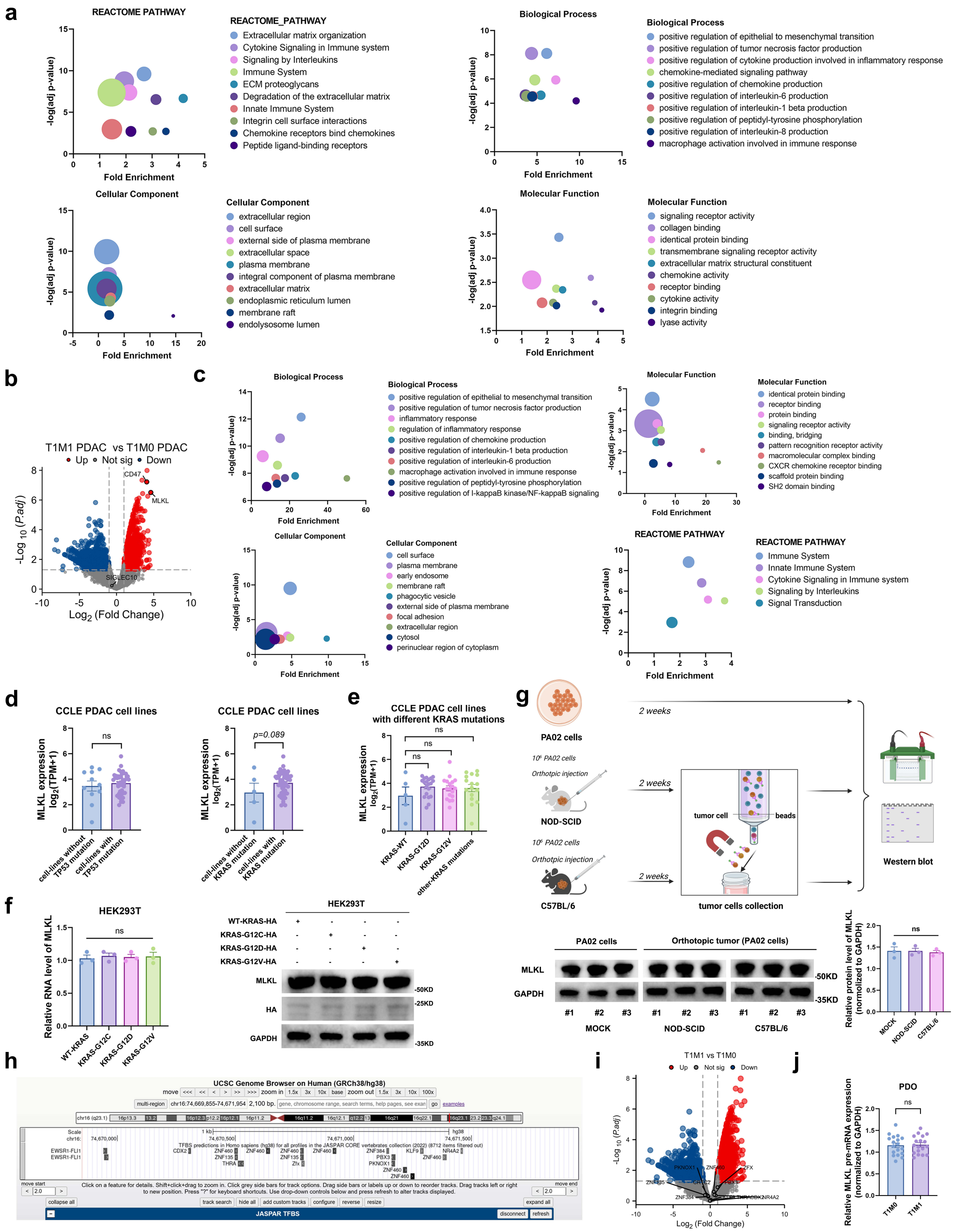

(a) REATCOME and GO pathway enrichment analyses of 2142 DEGs between T1M0-PDAC (n=6) and T1M1-PDAC samples (n=6).

(b) Volcano plot of the final 2142 DEGs between T1M0-PDAC (n=6) and T1M1-PDAC (n=6) samples.

(c) REATCOME and GO pathway enrichment analysis of the final 216 DEGs between T1M0-PDAC (n=6) and T1M1-PDAC (n=6) samples.

(d) MLKL expression of CCLE PDAC cell lines with different KRAS and TP53 mutations.

(e) MLKL expression of CCLE PDAC cell lines with different types of KRAS mutations.

(f) MLKL RNA and protein expression of HEK293T transfected with wild-type KRAS and different KRAS mutations; The RNA levels among different groups were analysed by using Kruskal-Wallis test; n=3 biologically independent samples.

(g) MLKL expression of different treated PA02 cells; Treatments were indicated as the flowchart; n=3 mice for each group. The MLKL expression levels among different groups were quantified and analysed by using Kruskal-Wallis test.

(h) Predicted transcriptional factors of MLKL according to JASPAR database.

(i) Predicted transcriptional factors and other previously-reported factors that regulating MLKL expression in RNA-seq data.

(j) MLKL pre-mRNA of T1M0-PDO (n=6) and T1M1-PDO (n=6); n=3 biologically independent samples.

Unless specified otherwise, the data are presented as means ± SEM (error bar), and compared using two-sided Student's *t* test; *, $P<0.05$; **, $P<0.01$; ***, $P<0.001$; ns, no significance. **f** is created with BioRender.com. Source data are provided as a Source Data file.

Supplementary Fig. 2 The association between survival and MLKL expression in PDAC.

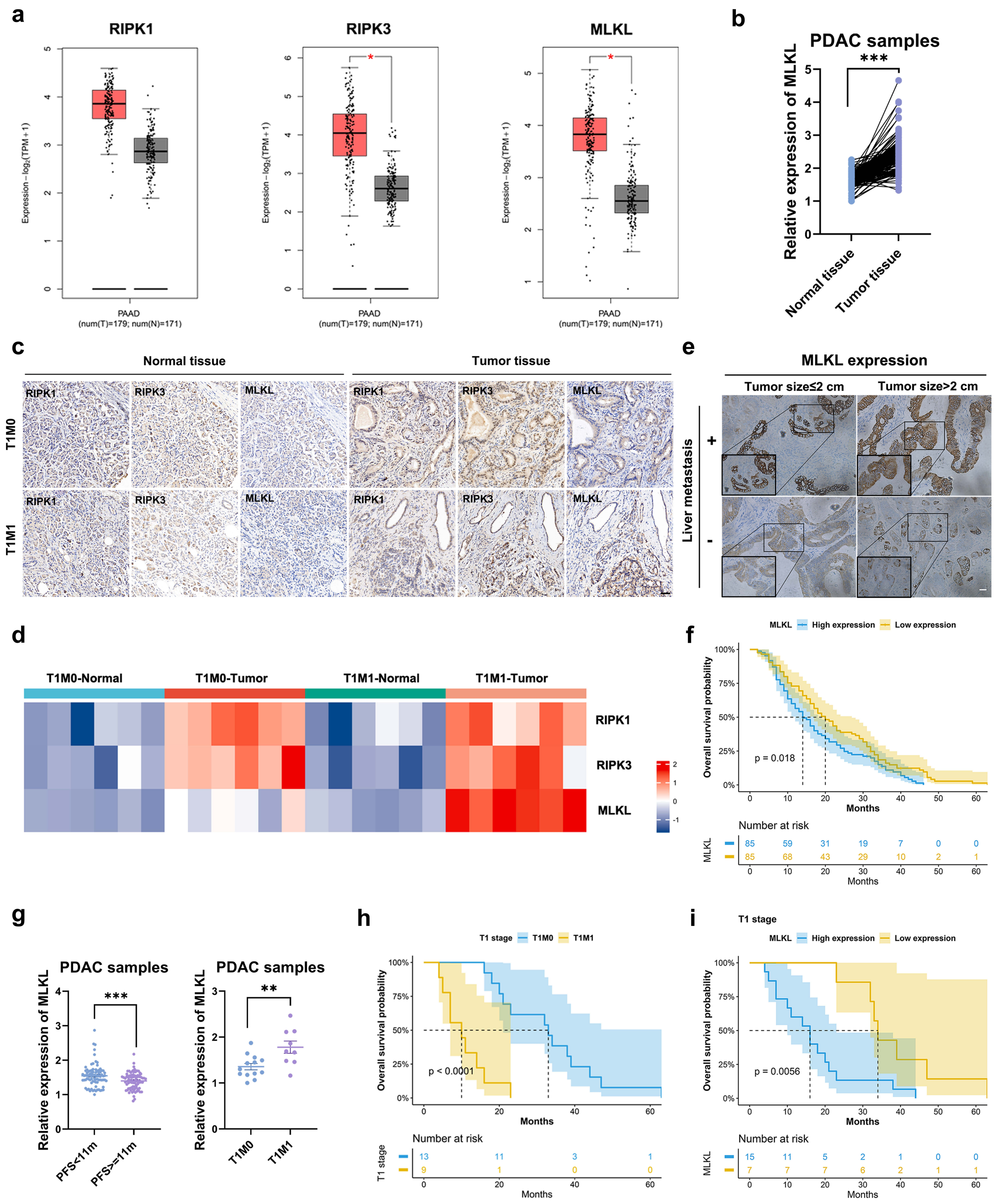

(a) MLKL/RIPK1/RIPK3 expression in PDAC tissues and normal tissues from PDAC patients in the TCGA database according to GEPIA2 online database.

(b) MLKL expression in PDAC tissues and normal tissues from PDAC patients at our centre (n=170); two-sided Student's *t* test.

(c) MLKL/RIPK1/RIPK3 expression in tumour tissue and normal tissue of T1M0-PDAC (n=6) and T1M1-PDAC (n=6) based on IHC. Scale bars: 50 μm.

(d) The RIPK1, RIPK3, and MLKL expression heatmap in tumour tissue and normal tissue of T1M0-PDAC (n=6) and T1M1-PDAC (n=6) according to the RNA-seq data.

(e) Expression of MLKL in representative primary PDAC tumours from our centre grouped according to tumour size (≤2 cm vs. >2 cm) and liver metastasis (+ or −); n=10 for each group; Scale bars: 20 μm.

(f) Kaplan–Meier survival curves of PDAC patients at our centre in the low MLKL expression group (n=85) and high MKLK expression group (n=85); two-sided log-rank test.

(g) MLKL expression in PDAC patients with a short PFS (n=83) and long PFS (n=87) at our centre; and MLKL expression in T1M0-PDAC (n=13) and T1M1-PDAC (n=9) patients at our centre; each dot represents a patient, and the median and percentile Inter-Quartile Range were shown as bold lines; two-sided Student's *t* test.

(h) Kaplan–Meier survival curves of OS in T1-stage PDAC patients at our centre and in T1M0 (n=13) and T1M1 PDAC (n=9) patients at our centre; two-sided log-rank test.

(i) Kaplan–Meier survival curves of OS in T1-stage PDAC patients at our centre stratified by MLKL expression into low MLKL expression (n=15) and high MLKL expression (n=7) groups; two-sided log-rank test;

*, P<0.05; **, P<0.01; ***, P<0.001; ns, no significance. **f** is created with BioRender.com. Source data are provided as a Source Data file.

Supplementary Fig. 3 The expression and activation mode of necroptosis.

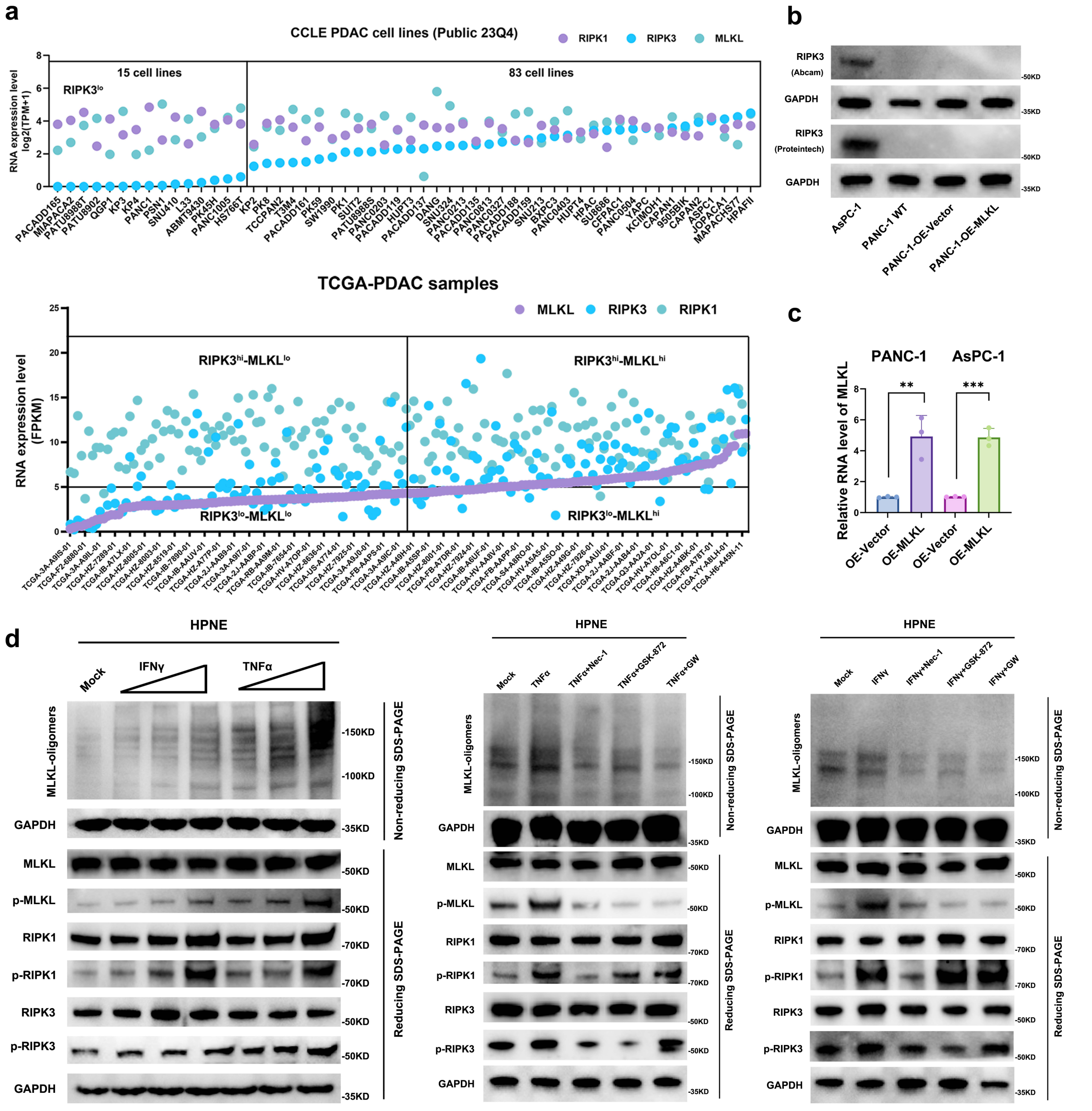

(a) RIPK1, RIPK3 and MLKL expression in CCLE PDAC cell lines and in TCGA PDAC data.

(b) RIPK3 protein level of PANC-1 cells detected by two antibodies; AsPC-1 was used for positive control cell; n=3 biologically independent samples.

(c) RT−PCR analysis of MLKL expression in PANC-1-OE-MLKL and AsPC-1-OE-MLKL cells; n=3 biologically independent samples; data are shown as mean±SEM and compared using two-sided Student's *t* test; **, *P*<0.01; ***, *P*<0.001; ns, no significance.

(d) WB analysis of HPNE necroptosis induced by TNFα, IFNγ and it could be rescued by RIPK3

inhibitor(GSK-872, 3 μM), as well as RIPK1 inhibitor (Nec-1, 50 μM) and MLKL inhibitor (GW, 1 μM); n=3 biologically independent samples. Source data are provided as a Source Data file.

Supplementary Fig. 4 The effects of MLKL knockout and mouse models.

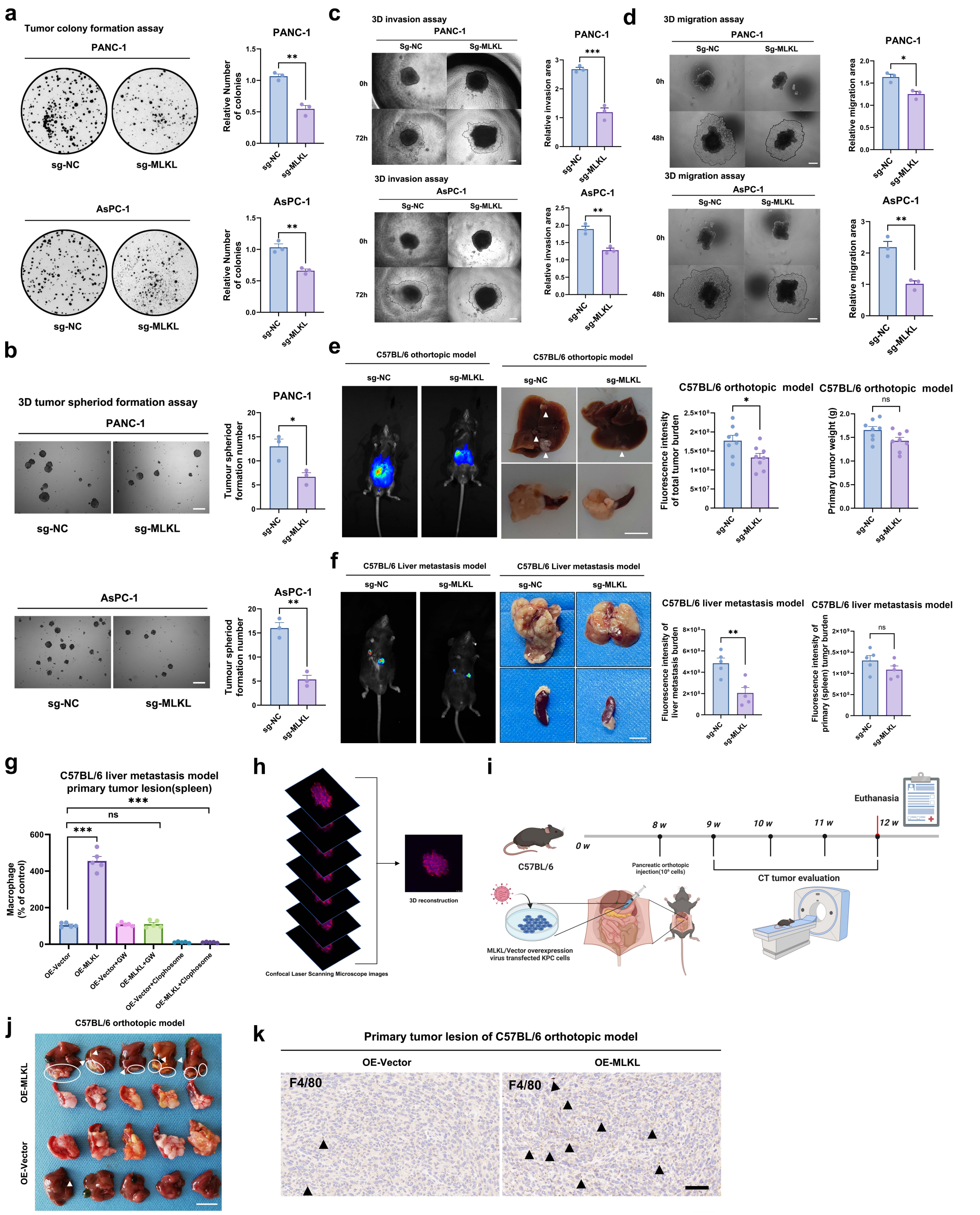

(a) 2D colony formation assays of control or MLKL-knockout PANC-1 and AsPC-1 cells; n=3 biologically independent samples.

(b) 3D tumour spheroid formation assays of control or MLKL-knockout PANC-1 and AsPC-1 cells; n=3 biologically independent samples

(c) 3D invasion assays of control or MLKL-knockout PANC-1 and AsPC-1 cells; n=3 biologically independent samples.

(d) 3D migration assays of control or MLKL-knockout PANC-1 and AsPC-1 cells; n=3 biologically independent samples.

(e) The C57BL/6 orthotopic model was generated by injecting control or MLKL-knockout KPC cells, n=8 mice for each group.

(f) The C57BL/6 liver metastasis model was generated by injecting control or MLKL-knockout KPC cells; n=5 mice for each group.

(g) Macrophages from the spleens of the C57BL/6 liver metastasis model mice after different treatments. The macrophages were quantified by flow cytometry as $CD45^+$ - $CD11b^+$ cells; n=5 mice for each group.

(h) The workflow used to 3D reconstruct F-actin staining images of tumour spheroids.

(i) The workflow used to generate C57BL/6 orthotopic models.

(j) Specimen from C57BL/6 mice orthotopically injected with Vector/MLKL overexpression; white circles and white arrows indicate liver-metastasis; Scale bars: 10 mm; n=5 mice for each group.

(k) IHC analysis of the macrophage marker F4/80 in primary tumour lesions of C57BL/6 orthotopic model. The triangles indicate F4/80-positive macrophages; n=5 mice for each group; Scale bar: 50 μm. All data are the mean±SEM and compared using Student's *t* test unless indicated; *, P<0.05; **, P<0.01; ***, P<0.001; ns, no significance. **i** is created with BioRender.com. Source data are provided as a Source Data file.

Supplementary Fig. 5 The macrophage and epithelial annotation of different clusters according to scRNA-seq.

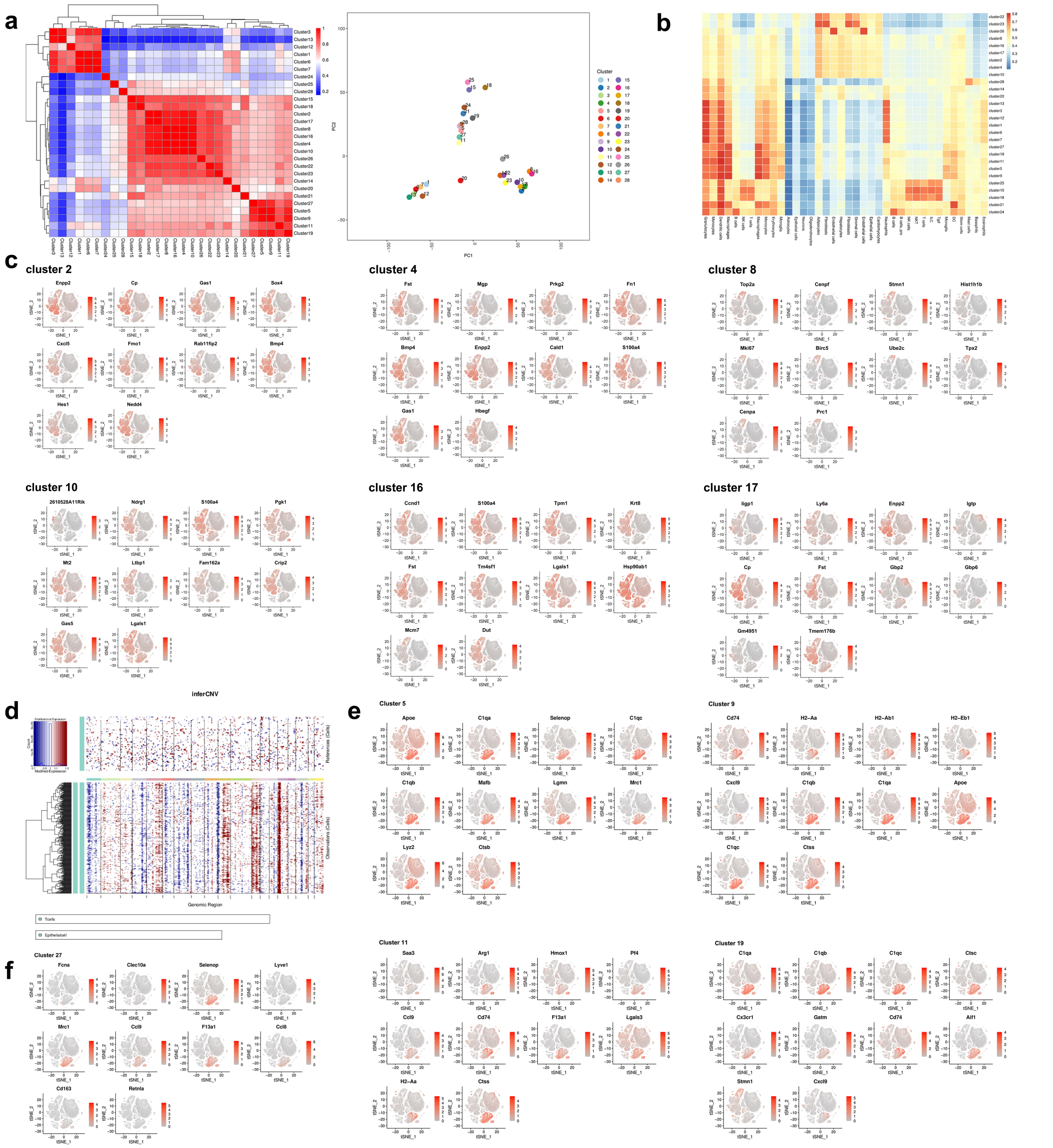

g

Name	Cluster	Maker gene
M1-macrophage	Cluster 1、2、5、6、11、13	*H2-Aa*、*H2-Ab1*、*H2-Eb1*、*TNF*、*Stat1*
M2-macrophage	Cluster 3、4、8、10、12	*MRC1*、*Arg1*、*Il1r2*、*Chil3*、*Tgfb1*
lipid-associated macrophage	Cluster 9	*Apoe*、*Apoc*
CXCL9+ macrophage	Cluster 14、18	*CXCL9*
Immunomodulatory macrophage	Cluster 15	*CXCL1*、*S100A9*、*S100A10*、 *CCL2*、 *CCL6*、 *CCL9*、*Cxcl5 CCR2*、*CXCL13*、*CCL12*、*CXCR4*
SPP1+ macrophage	Cluster 16	*SPP1*
C3+ macrophage	Cluster 17	*C3*、*Ifit1*、*Ifit2*、*Ifit3*
CD8+ T cell-like macrophage	Cluster 19	*GZMA*、*GZMB*、*CD8A*、 *CD27*

(a) A correlation heatmap and PCA map of the different clusters of cells that harvest from the orthotopic tumours in OE-Vector group and OE-MLKL group of orthotopic models (3 mice per group).

(b) The heatmap shows the results of SingleR prediction based on the clustering results. Each column represents a cell type in the database, and each row represents a clustered cell group (cluster); The colour represents the Spearman correlation coefficient score, with increasing amounts of red indicating a greater likelihood that a certain cell cluster is a certain cell type.

(c) Individual cells (points) in two-dimensional t-SNE plots are colour-coded in shades of red according to the levels of cell type-specific gene signatures that were used to characterize and annotate the cancer cell cluster populations.

(d) Copy number variant analysis.

(e) Individual cells (points) in two-dimensional t-SNE plots are colour-coded in shades of red according to the levels of cell type-specific gene signatures that were used to characterize and annotate the macrophage cluster populations.

(f) Individual cells (points) in two-dimensional t-SNE plots are colour-coded in shades of red according to the levels of cell type-specific gene signatures that were used to characterize and annotate the macrophage cluster populations.

(g) Marker genes used to classify macrophage type.

Supplementary Fig. 6 MLKL-driven necroptosis recruits and activates macrophages.

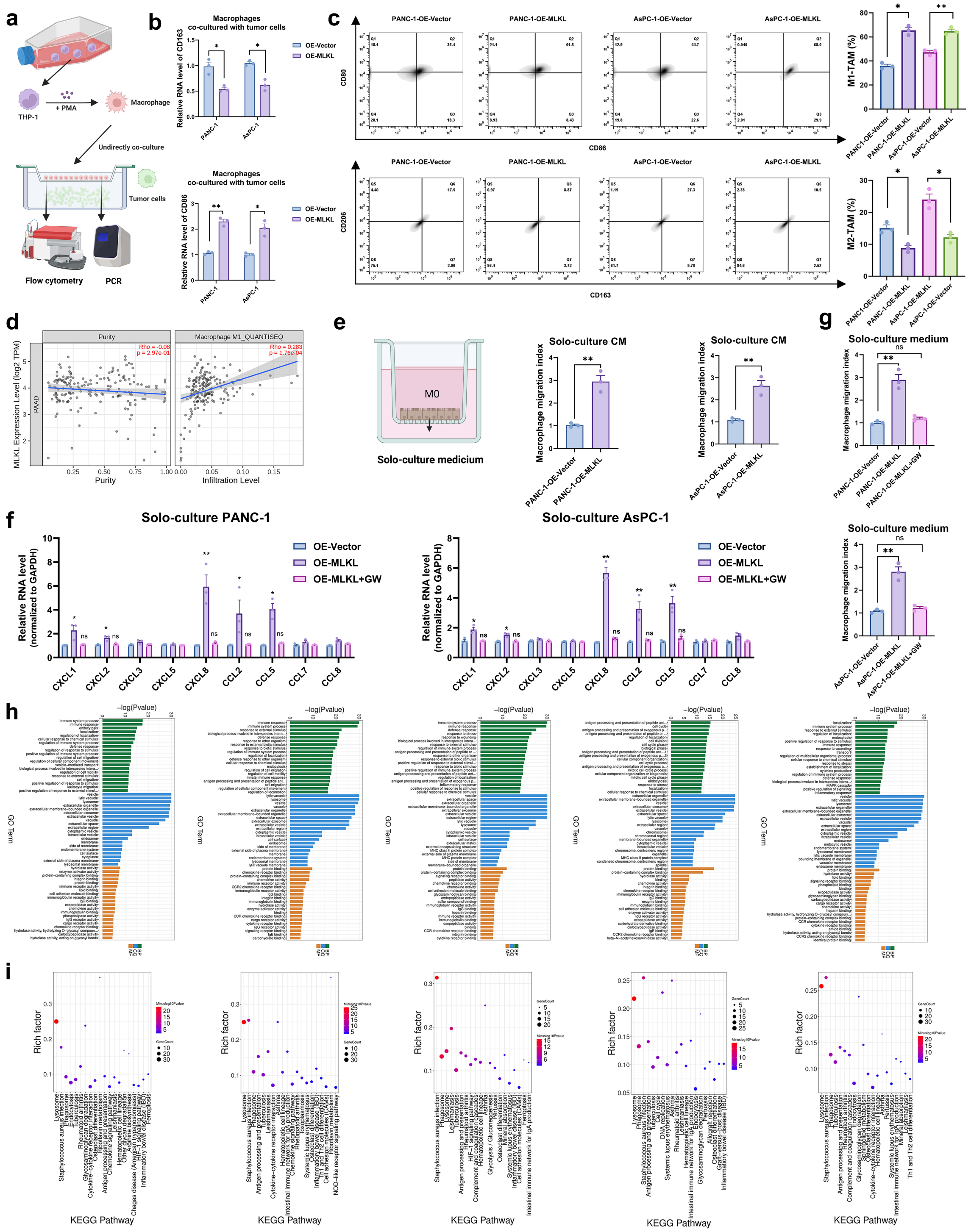

(a) The workflow used to co-culture the macrophages.

(b) The RNA levels of CD86 and CD163 in macrophages co-cultured with PDAC cells; n=3 biologically independent samples.

(c) Polarization analysis of macrophages type after co-culture with PDAC cells via flow cytometry; n=3 biologically independent samples.

(d) Correlations analysis between the MLKL expression level and M1 macrophage infiltration in PDAC patients through online TIMER database according to TAGA-PDAC data based on QuanTIseq method.

(e) The workflow and effects of the coincubation of macrophages with the CM supernatant from PDAC cells on migration; n=3 biologically independent samples.

(f) The RNA levels of macrophage chemokines in PDAC cells determined via qPCR; n=3 biologically independent samples.

(g) The migration capacity of macrophages treated with different PDAC cell culture supernatants was analysed; n=3 biologically independent samples.

(h) GO enrichment analysis of the macrophage clusters.

(i) KEGG enrichment analysis of the macrophage clusters.

All data are the mean±SEM and compared using Student's *t* test unless indicated; *, P<0.05; **, P<0.01; ***, P<0.001; ns, no significance. **a** and **e** created with BioRender.com. Source data are provided as a Source Data file.

Supplementary Fig. 7 The measurement of macrophage phagocytosis events and the upregulation of CD47 through IL6.

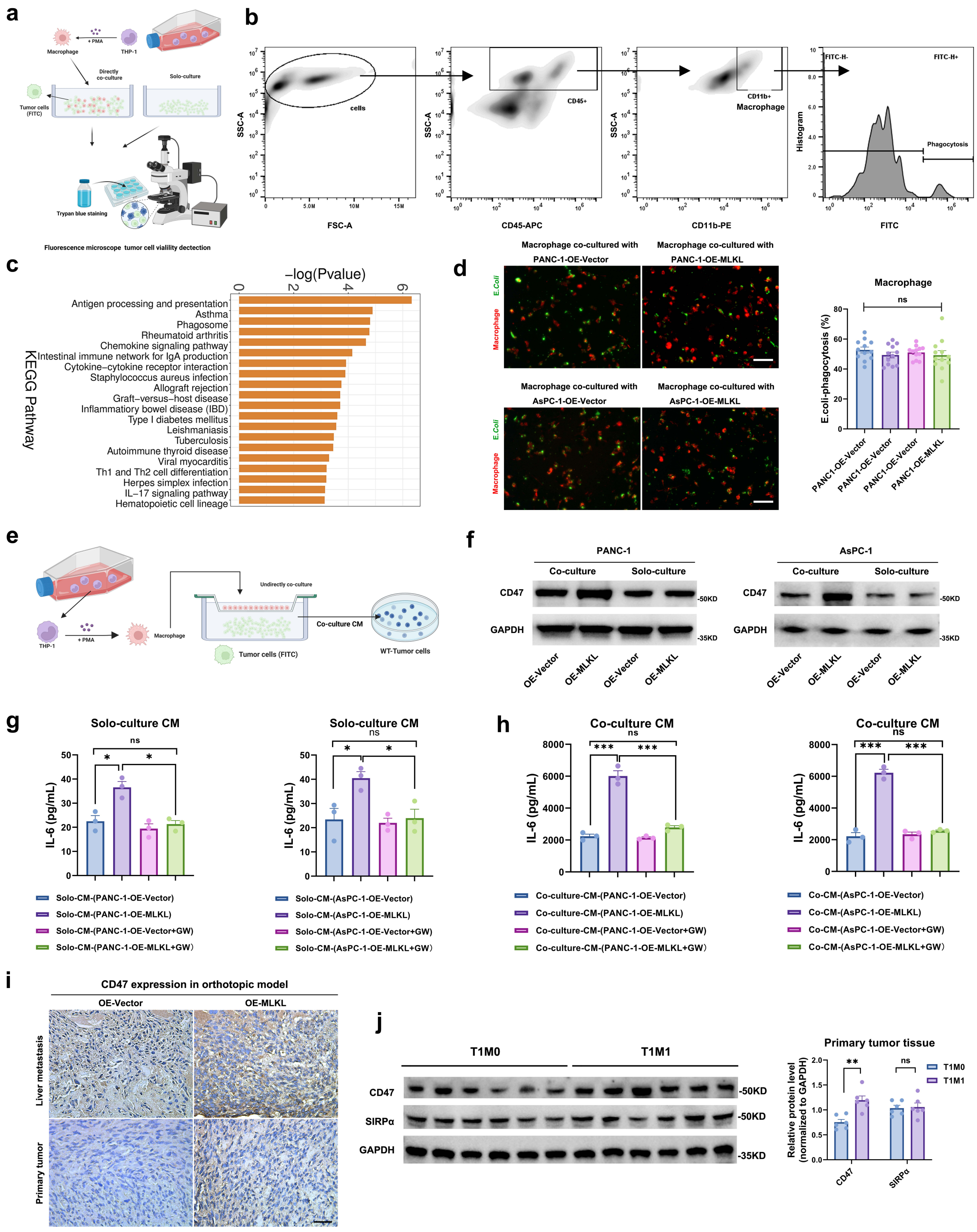

(a) Diagram of the cell viability assay.

(b) Flow cytometry gating strategy used to identify THP-1-derived macrophages ($CD45^{+}$ - $CD11b^{+}$) and tumour phagocytosis events ($FITC^{+}$), which correspond to Fig 4e-4f. The cells were selected by size using SSC-A or FSC-A; CD11b was gated to separate the macrophages, and FITC was subsequently gated to detect uptake by the macrophages.

(c) KEGG analysis of scRNA-seq data from macrophages from mouse tumours overexpressing the vector or MLKL.

(d) Representative immunofluorescence images showing the phagocytosis of labelled *E. coli* (green) by DiI-labelled macrophages (red) co-cultured with tumour cells; The arrows indicate phagocytic events that were assessed via fluorescence microscopy; Four random fields were assessed in each sample; The difference of phagocytic events among different groups was analysed by using Kruskal-Wallis test; n=3 biologically independent samples; Scale bar, 100 μm.

(e) Workflow showing the treatment of wild-type tumour cells with co-culture CM.

(f) WB analysis of CD47 in control and PANC-1-OE-MLKL cells cultured alone or co-cultured with macrophages; n=3 biologically independent samples;

(g) ELISA of IL-6 in CM from solocultures after different treatments; n=3 biologically independent samples;

(h) ELISA of IL-6 in CM collected following co-culture with different treatments; n=3 biologically independent samples;

(i) IHC staining for CD47 in primary tumours and liver metastases in the orthotopic model; n=5 mice for each group.

(j) CD47 and SIRPα levels in T1M0-PDAC (n=6) and T1M1-PDAC (n=6) tumour tissues.

All data are the mean±SEM, and compared using Student's *t* test unless indicated; *, $P<0.05$; **, $P<0.01$; ***, $P<0.001$; ns, no significance. **a** and **e** are created with BioRender.com. Source data are provided as a Source Data file.

Supplementary Fig. 8 MLKL-driven necroptosis trigger METs formation through Supernatant.

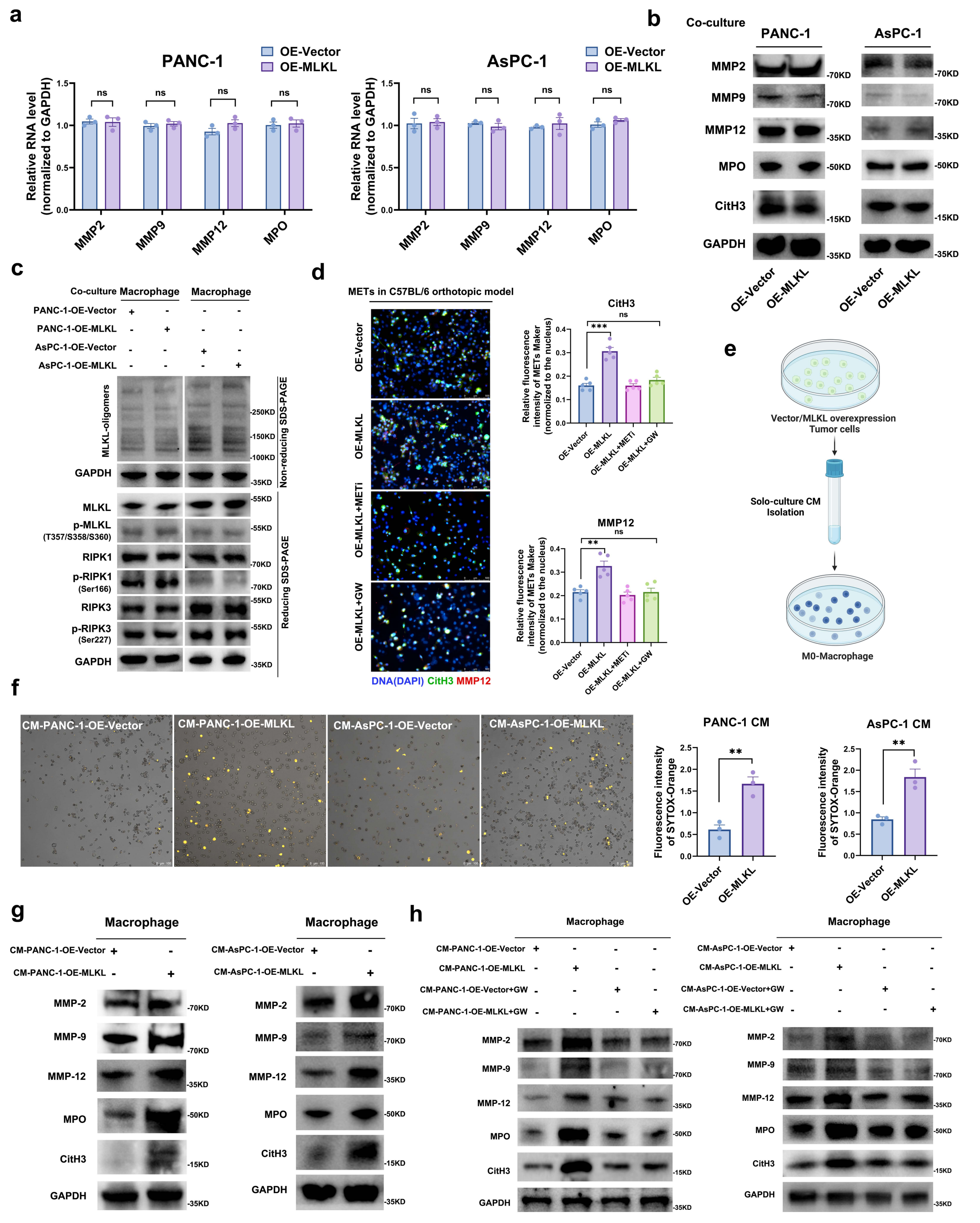

(a) The RNA levels of MMP2, MMP9, MMP12 and MPO in co-cultured tumour cells; n=3 biologically independent samples.

(b) The protein levels of MMP2, MMP9, MMP12 and MPO in co-cultured tumour cells; n=3 biologically independent samples.

(c) WB analysis of necroptosis in macrophages co-cultured with tumour cells; n=3 biologically independent samples.

(d) Representative immunostaining of METs (green, CitH3; red, MMP12) and DNA (blue, DAPI) in primary tumours from C57BL/6 orthotopic model mice administered the indicated treatment. GW (100 µM in 50 µl, iv., 3×/week) or MET inhibitor (Cl-amidine, 50 mg/kg, iv., 3×/week) was administered. n=5 mice in each group. The mice were euthanized 4 weeks after injection. Scale bar: 500 µm.

(e) Diagram of macrophages treated with CM from tumour cells cultured alone.

(f) SYTOX Orange staining and quantification of METs in macrophages treated with CM from a monoculture or CM following co-culture with tumour cells; n=3 biologically independent samples.

(g) WB analysis of MET-related proteins (CitH3, MPO, MMP-2, MMP-9, and MMP12) in macrophages treated with supernatant derived from tumour cells. The macrophages were collected together with the CM as the sample; n=3 biologically independent samples.

(h) WB analysis of MET-related proteins (CitH3, MPO, MMP-2, MMP-9, and MMP12) in macrophages treated with supernatant derived from tumour cells treated with or without GW; n=3 biologically independent samples.

All data are the mean±SEM, and compared using Student's *t* test; *, P<0.05; **, P<0.01; ***, P<0.001; ns, no significance. **e** is created with BioRender.com. Source data are provided as a Source Data file.

Supplementary Fig. 9 MLKL-driven necroptosis releases CXCL8 to trigger METs formation.

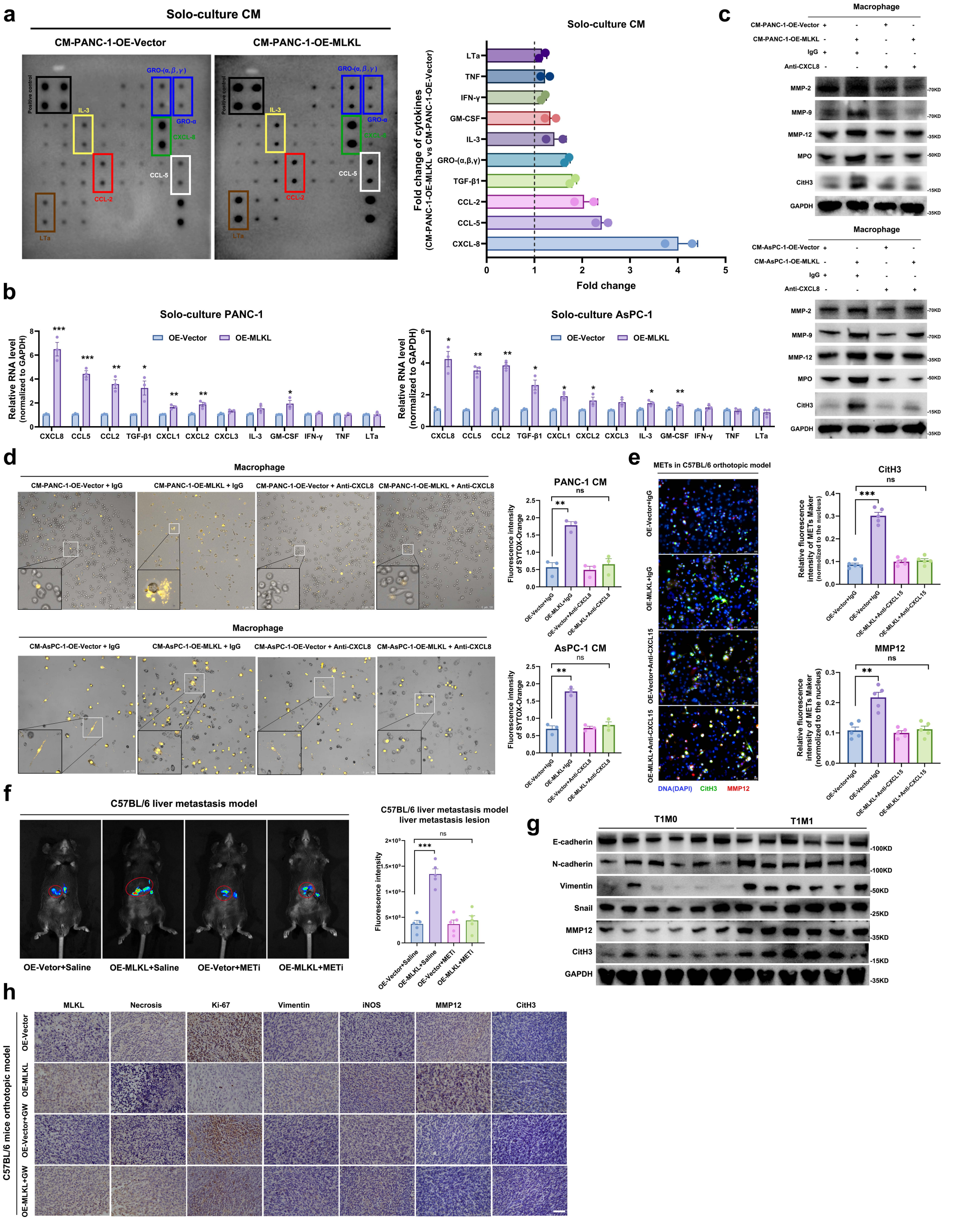

(a) Representative human cytokine antibody array and the corresponding quantitation (right) of CM following solo-culture; n=3 biologically independent samples.

(b) PCR analysis of cytokines of CM following solo-culture; n=3 biologically independent samples.

(c) WB analysis of MET-related proteins (CitH3, MPO, MMP-2, MMP-9, and MMP12) in macrophages in the presence of CM from tumour cells cultured alone. Treatments were administered (IgG, 200 μg; anti-CXCL8, 200 μg). The samples were ultimately collected together with the CM.

(d) SYTOX Orange staining and quantification of MET-related extracellular DNA in macrophages treated with CM from tumour cells cultured alone. Treatments were administered (IgG, 200 μg; anti-CXCL8, 200 μg); n=3 biologically independent samples.

(e) Representative immunostaining of METs (green, citrullinated histone H3 [cit-H3], red, MMP12) and DNA (blue, DAPI) in the primary tumours of C57BL/6 orthotopic model mice subjected to the corresponding treatment (IgG, anti-CXCL15 [CXCL15 is the mouse homologue of human CXCL8]; 200 μg, 3×/week). Orthotopic tumours were harvested at four weeks after injection; n=5 mice for each group; Scale bar: 500 μm.

(f) Bioluminescence of liver metastases and primary spleen nodes of mice in the different groups treated with saline, METi (Cl-amidine, 50 mg/kg, iv., 3×/week) at three weeks after the injection of one million OE-vector/MLKL cells into the spleen. Red circle indicated the bioluminescence of liver metastasis burden; n=5 mice for each group;

(g) The protein levels of EMT and MET markers in T1M0-PDAC (n=6) and T1M1-PDAC (n=6) tissues.

(h) IHC analysis of markers of proliferation, EMT, macrophages and METs in primary tumours from the C57BL/6 orthotopic model mice after different treatments were applied; n=5 mice for each group; Scale bar:50 μm.

All data are the mean±SEM, and compared using Student's *t* test unless indicated; *, P<0.05; **, P<0.01; ***, P<0.001; ns, no significance. Source data are provided as a Source Data file.

Supplementary Fig. 10 CXCL8 promotes EMT and upregulates ICAM1 in tumour cells.

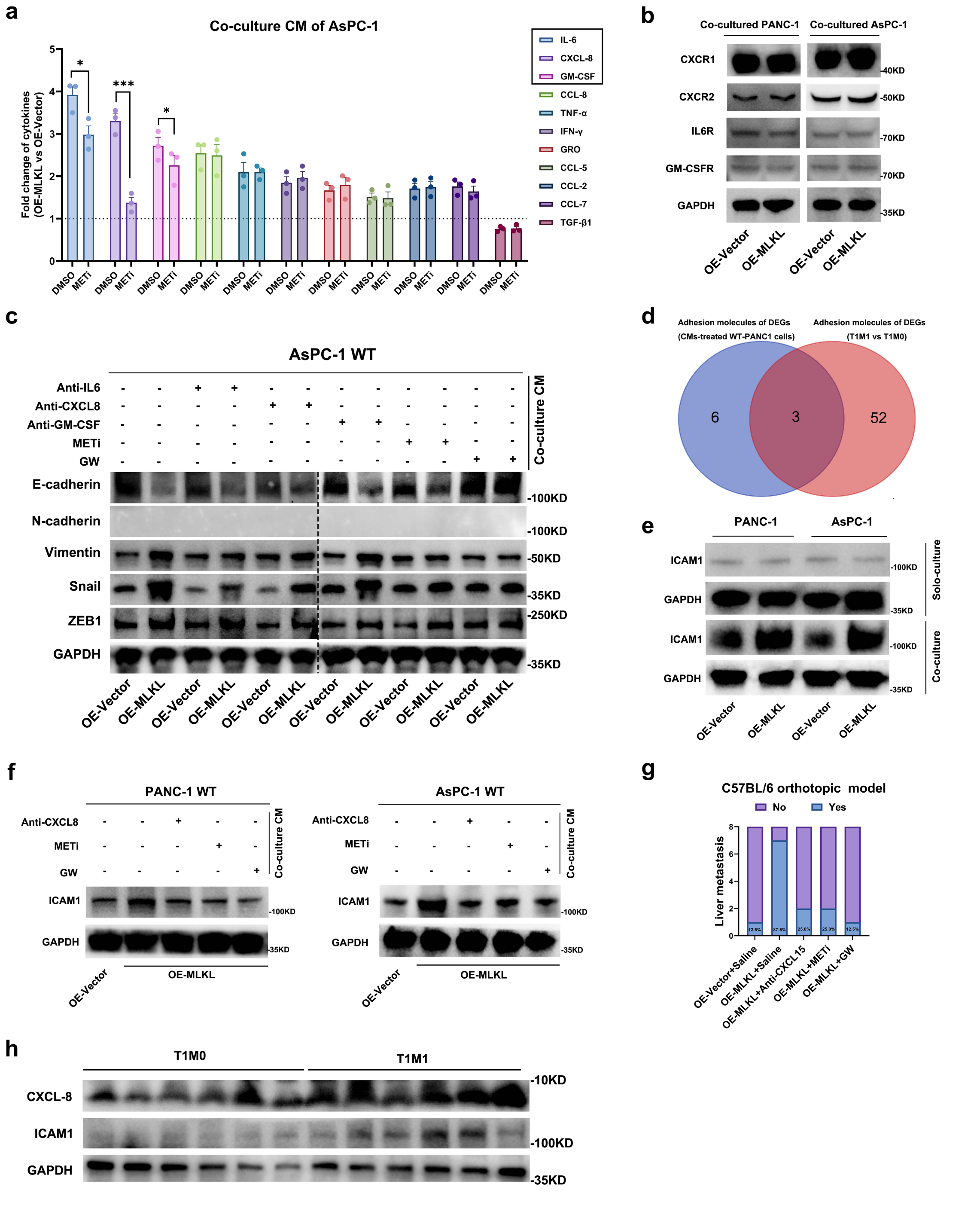

(a) ELISA of cytokines after co-culture with CM and treatment with DMSO or a MET inhibitor (Cl-amidine); n=3 biologically independent samples.

(b) WB analysis of CXCR1, CXCR2, IL6R and GM-CSFR in tumour cells co-cultured with

macrophages; n=3 biologically independent samples.

(c) WB analysis of EMT markers in wild-type tumour cells treated with different types of CMs collected after co-culture (anti-IL6, 200 μg; anti-GM-CSF, 200 μg; anti-CXCL8, 200 μg; a MET inhibitor, Cl-amidine, 25 μg/ml; GW, 1 μM) for 48 hours; n=3 biologically independent samples.

(d) Changes in adhesion molecules (CDH1, CDH2, and ICAM1) in wild-type PANC1 cells treated with different CMs or T1M0 and T1M1 PDAC samples determined according to RNA-seq data.

(e) WB analysis of ICAM1 in tumour cells cultured alone or co-cultured with macrophages; n=3 biologically independent samples.

(f) WB analysis of ICAM1 in wild-type tumour cells that received different types of CM (CM collected after CXCL8 was neutralized, CMs derived from co-culture experiments with a METi or GW); n=3 biologically independent samples.

(g) Incidence of liver metastasis in the C57BL/6 orthotopic model mice after treatment with saline, anti-CXCL15 (200 μg, iv., 3×/week), GW (100 μM in 50ul, iv. 3×/week), or a MET inhibitor (Cl-amidine, 50 mg/kg, iv., 3×/week). n=8 in each group. The mice were sacrificed 4 weeks after injection.

(h) WB analysis and quantification of the protein levels of ICAM1 and CXCL8 in T1M0-PDAC (n=6) and T1M1-PDAC (n=6) tumour tissues.

All data are the mean±SEM, and compared using Student's *t* test unless indicated; *, P<0.05; **, P<0.01; ***, P<0.001; ns, no significance. Source data are provided as a Source Data file.

Supplementary Fig. 11 METs create an ECM-degrading niche.

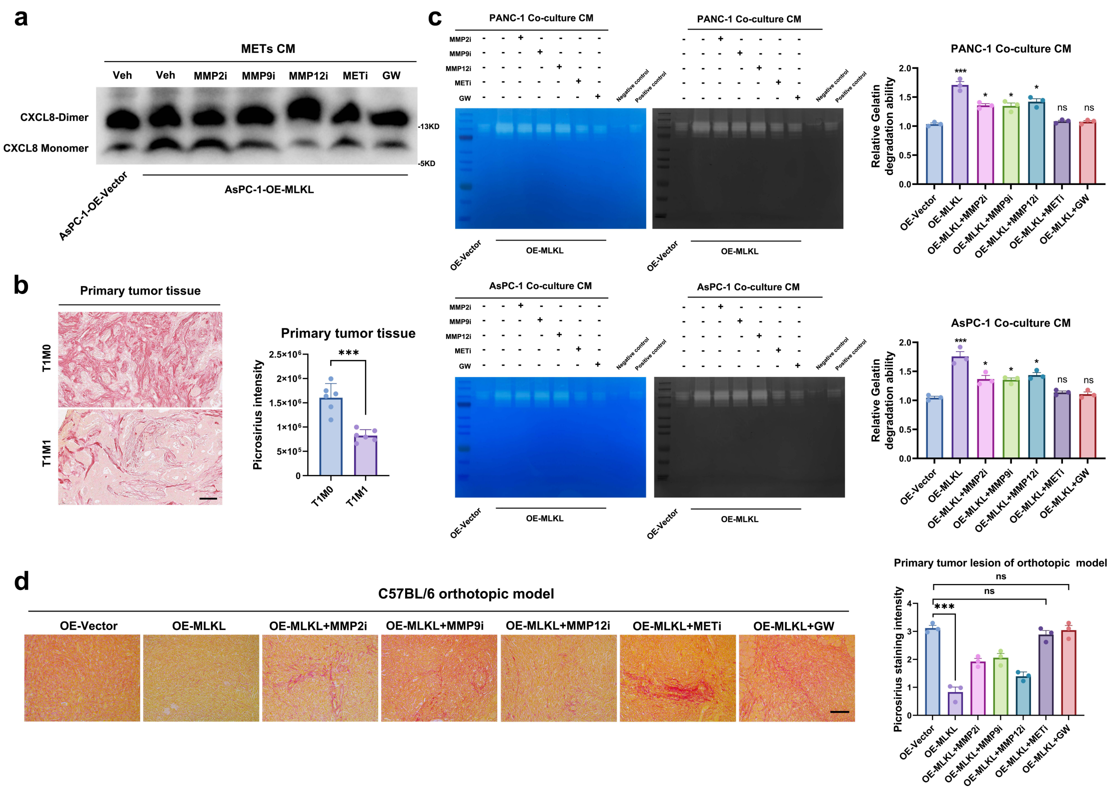

(a) WB analysis of the CXCL8 dimer and monomer from the MET-containing CM following different treatments, 20% nonreducing SDS–PAGE was used to separate the proteins; n=3 biologically independent samples.

(b) Picrosirius staining of primary tumours from T1M1-PDAC (n=6) and T1M0-PDAC (n=6) samples and quantification of the data. Scale bar: 100 μm.

(c) Gelatine zymography analysis of CM obtained after co-culture with different treatments. A white blot indicates digested gelatine, reflecting the ability to degrade ECM; Relative gelatine degradation ability of each group was quantified and compared with that in the OE-Vector group, n=3 biologically independent samples.

(d) Picrosirius staining of primary tumours from orthotopic model mice after different treatments and quantification of the results. n=3 in each group. Scale bar: 100 μm.

All data are the mean±SEM, and compared using Student's *t* test unless indicated; *, P<0.05; **, P<0.01; ***, P<0.001; ns, no significance. Source data are provided as a Source Data file.

Supplementary Fig. 12 Construction and validation of MLKL-overexpressing KDOs.

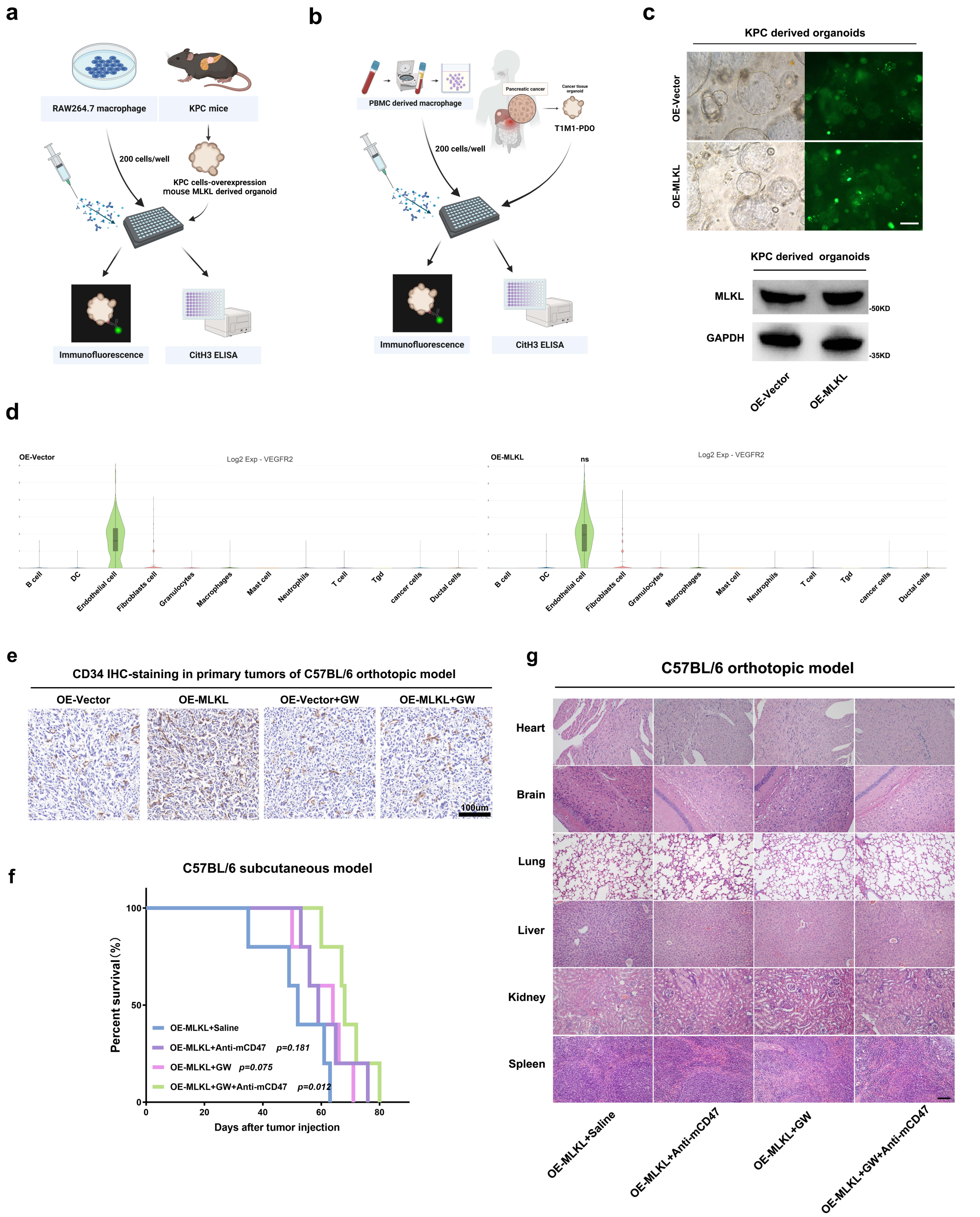

(a) Method used to co-culture KPC mouse-derived organoids overexpressing MLKL (KDOs) and RAW264.7 macrophages with the application of different treatments and quantitation of the organoid volume and E-cadherin fluorescence.

(b) The method used for the co-culture of T1M1 PDAC PDOs and PBMC-derived macrophages with the application of different treatments and the quantitation of organoid volume and E-cadherin fluorescence.

(c) Overexpression of murine MLKL in KPC-derived organoids and WB analysis of MLKL in MLKL-overexpressing KDOs; n=3 biologically independent sample; Scale bar: 100 µm.

(d) The expression of VEGFR2 of orthotopic tumours in OE-Vector and OE-MLKL group based on the scRNA-seq data; ns indicated that there is no statistically significant difference of VEGFR2 expression in endothelial cells between the two groups.

(e) The IHC staining of vessels maker CD34 of orthotopic tumours in OE-Vector and OE-MLKL group in C57BL/6 orthotopic model; n=5 mice for each group; Scale bar: 100 µm.

(f) Mouse Kaplan-Meier survival curves (two sided log-rank test); n=5 mice for each group.

(g) Representative H&E microscopy images of major organs from the different groups of C57BL/6 orthotopic model mice; 8 mice per group; Scale bar: 100 µm.

a and **b** are created with BioRender.com. Source data are provided as a Source Data file.

Supplementary Fig.13 Graphical Abstract of proposed mechanism through which necroptosis promotes liver metastasis in PDAC.

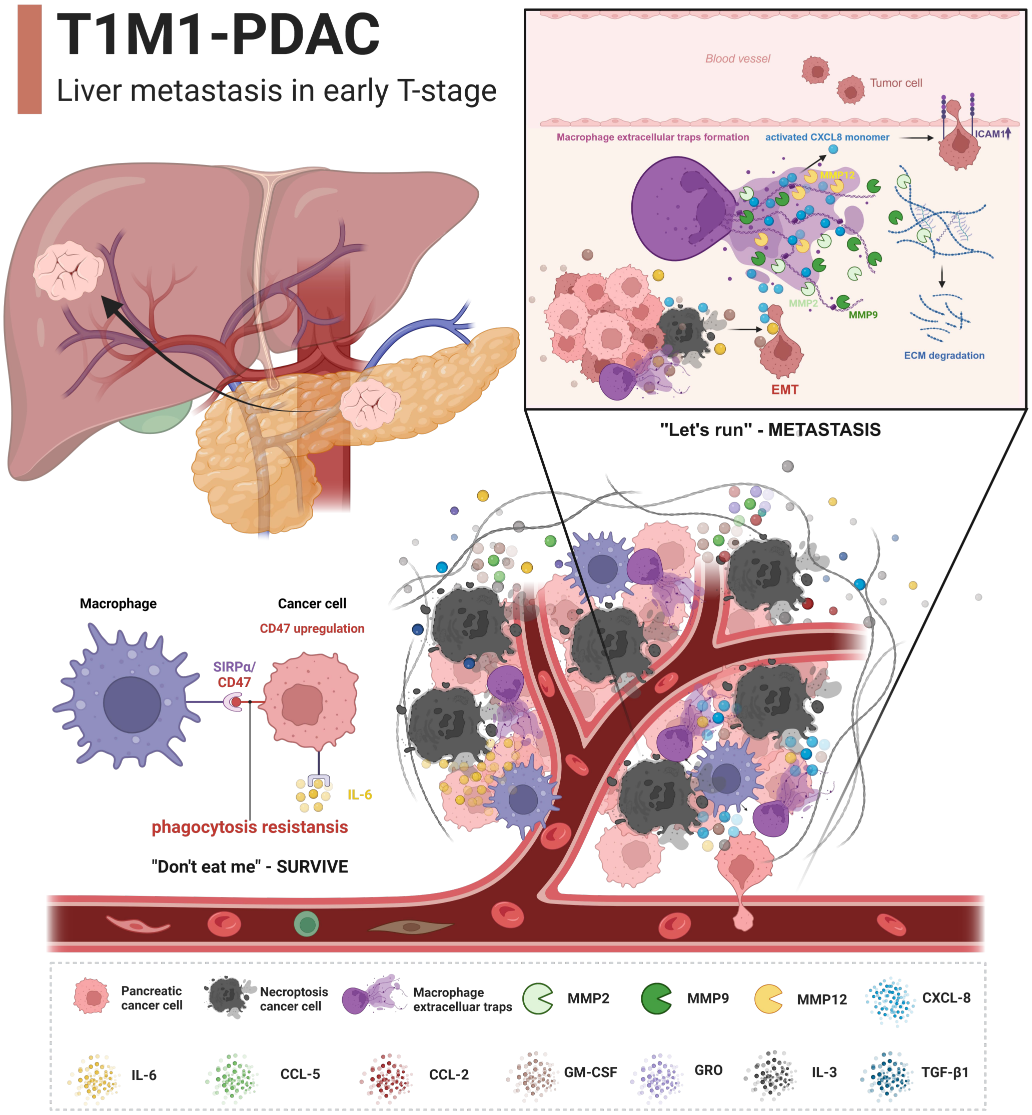

MLKL-driven necroptosis releases cytokines, which leads to the recruitment of macrophages and their differentiation into M1 macrophages. Then, the large amount of IL-6 in the TME upregulates CD47 expression in neighbouring tumour cells, thus increasing resistance to phagocytosis. Necroptosis also promotes the release of CXCL8, inducing the formation of METs, which then cleave CXCL8 into the activated CXCL8 monomer. The activated CXCL8 monomer promotes EMT and ICAM upregulation in tumour cells. METs also promote the degradation of the ECM and create a pro-metastatic niche. Additionally, METs promote the three steps of liver metastasis: EMT initiation, ECM degradation, and endothelial adhesion. In this context, tumours behave in a "don't

eat me, let's run” signal that promotes metastasis to the liver. **Highlights:** (1) Necroptosis hijacks macrophages and induces macrophage extracellular traps (MET) formation to create a metastatic niche, revealing a new mechanism of liver metastasis in early T-stage PDAC. (2) Necroptosis enhances the tumour “don’t eat me, let's run” signal for immune evasion and liver metastasis. (3) METs trap CXCL8 and cleave CXCL8 into an activated CXCL8 monomer. (4) METs play important roles in multiple steps of metastasis, initiating EMT, ECM degradation, and endothelial adhesion. (5) The combination of necroptosis inhibitor treatment and CD47 blockade provides a novel neoadjuvant immunotherapy solution for the clinical dilemma of liver metastasis in early T-stage PDAC to regain the opportunity for radical surgery.

Supplementary Tables

Supplementary Table 1. Details of enrolled PDAC patients who received magnetic resonance imaging.

No.	Group	Age	Sex	CA-199 U/mL	KRAS mutation	TP53 mutation	Primary tumour	Differentiation	Primary tumour size, cm	Primary tumour location	liver metastasis	liver metastasis size, cm	liver metastasis location
P1	T1M0	67	female	710.5	Yes	Yes	single	moderate	1.9*1.9	body/tail	/	/	/
P2	T1M0	68	female	198	Yes	Yes	single	moderate	1.5*2.0	head	/	/	/
P3	T1M0	58	male	48.18	No	Yes	single	poor	1.8*2.0	body/tail	/	/	/
P4	T1M0	73	female	38.52	Yes	Yes	single	moderate	1.4*1.3	head	/	/	/
P5	T1M0	56	male	8.27	No	No	single	moderate	1.5*1.4	head	/	/	/
P6	T1M0	74	male	383.6	Yes	Yes	single	moderate	1.9*1.5	head	/	/	/
P7	T1M1	50	male	18.28	No	Yes	single	poor	1.7*2.0	head	multiple	1.3	S6/7
P8	T1M1	67	male	731.8	Yes	Yes	single	moderate	1.9*2.0	body/tail	multiple	1.4	S7/8
P9	T1M1	53	male	24.67	No	No	single	poor	1.4*2.0	body/tail	single	2.7	S7
P10	T1M1	65	male	332.77	Yes	Yes	single	poor	1.5*1.8	head	single	1.2	S7
P11	T1M1	71	female	760.2	Yes	Yes	single	poor	2.0*1.7	body/tail	multiple	3.8	S2/3/4/5/6/7/8
P12	T1M1	77	male	150.5	No	Yes	single	poor	1.8*1.4	body/tail	multiple	1.4	S2/3/4/5/6/7/8

Sex (biological attribute)

Supplementary Table 2. Baseline characteristics of MLKL in clinical PDAC samples.

Characteristics	Low expression MLKL n=85	High expression MLKL n=85	*p*
Age			0.219
<60	48(56.5)	40(47.1)	
≥60	37(43.5)	45(52.9)	
Sex			0.342
female	35(41.2)	29(34.1)	
male	50(58.8)	56(65.9)	
Tumor size			0.003
≤2cm	10(11.8)	26(30.6)	
>2cm	75(88.2)	59(69.4)	
Lymph node metastasis			0.080
no	27(31.8)	17(20.0)	
yes	58(68.2)	68(80.0)	
Liver metastasis			0.006
no	78(91.8)	65(76.5)	
yes	7(8.2)	20(23.5)	
Differentiation			0.014
poor	37(43.5)	53(62.4)	
well/moderate	48(56.5)	32(37.6)	
AJCC stage			0.042
I	18(21.2)	14(16.5)	
II	33(38.8)	24(28.2)	
III	27(31.8)	27(31.8)	
IV	7(8.2)	20(23.5)	

Sex (biological attribute); Pearson's χ^2 test, two-sided.

Supplementary Table 3. Primers.

Name	Sequence	Name	Sequence
MLKL-F(homo)	GCTCTCGCTGTTACTTCAGGT	IL-8-F(homo)	TGGCAGCCTTCCTGATTTCTG
MLKL-R(homo)	TGTCTTCGTCTGCATCCTGC	IL-8-R(homo)	ATTTGGGGTGGAAAGGTTTGG
GAPDH-F(homo)	GGTGTGAACCATGAGAAGTATGA	IL-10-F(homo)	AGCTGAGAACCAAGACCCAGA
GAPDH-R(homo)	GAGTCCTTCCACGATACCAAAG	IL-10-R(homo)	ACTCATGGCTTTGTAGATGCCTT
G-CSF-F(homo)	CTGCTTGAGCCAACTCCATAG	IL-13-F（homo)	TGGTATGGAGCATCAACCTGAC
G-CSF-R(homo)	GCCATTCCCAGTTCTTCCAT	IL-13-R（homo)	AGAATCCGCTCAGCATCCTC
GM-CSF-F(homo)	TCCTGAACCTGAGTAGAGACAC	IL-15-F(homo)	GTGATGTTCACCCCAGTTGC
GM-CSF-R(homo)	TGCTGCTTGTAGTGGCTGG	IL-15-R(homo)	CTCCAGTTCCTCACATTCTTTGC
GRO alpha-F(homo)	GGGAATTCACCCCAAGAACATC	IFN-gamma-F(homo)	AGGTCATTCAGATGTAGCGGATA
GRO alpha-R(homo)	GGATGCAGGATTGAGGCAAGC	IFN-gamma-R(homo)	TTCCTTGATGGTCTCCACACT
GRO beta-F(homo)	ATTCACCTCAAGAACATCCAAAGT	MCP-1-F(homo)	CAGCAAGTGTCCCAAAGAAGC
GRO beta-R(homo)	GACAAGCTTTCTGCCCATTCT	MCP-1-R(homo)	TCGGAGTTTGGGTTTGCTTG
GRO gamma-F(homo)	AACCGAAGTCATAGCCACACTC	MCP-2-F(homo)	GCTCAGCCAGATTCAGTTTCC
GRO gamma-R(homo)	GGTGCTCCCCTTGTTCAGTATC	MCP-2-R(homo)	AGATCACAGCTTCCTTGGGAC
IL-1alpha-F(homo)	CTGGGAAACTCACGGCACTA	MCP-3-F(homo)	GCTGAGACCAAACCAGAAACC
IL-1alpha-R(homo)	ACGCCTGGTTTTCCAGTATCT	MCP-3-R(homo)	GAAGTATTAATCCCAACTGGCTG
IL-2-F(homo)	CACCAGGATGCTCACATTTAAG	MIG-F(homo)	CCTCTTGGGCATCATCTTGCT
IL-2-R(homo)	CTCCAGAGGTTTGAGTTCTTCT	MIG-R(homo)	GATAGTCCCTTGGTTGGTGCT
IL-3-F(homo)	ACAACCTCAATGGGGAAGACC	RANTES-F(homo)	CCTCGCTGTCATCCTCATTG
IL-3-R(homo)	GATTGGATGTCGCGTGGGT	RANTES-R(homo)	CACTTGCCACTGGTGTAGAA
IL-5-F(homo)	CTCTTGGAGCTGCCTACGTG	TGF-beta1-F(homo)	GAGCCCTGGACACCAACTAT
IL-5-R(homo)	CCAGTGTGCCTATTCCCTGA	TGF-beta1-R(homo)	AAGTTGGCATGGTAGCCCTT
IL-6-F(homo)	AAGCCAGAGCTGTGCAGATG	TNF-alpha-F(homo)	TCCTCTCTGCCATCAAGAGC
IL-6-R(homo)	CTGGCATTTGTGGTTGGGTC	TNF-alpha-R(homo)	AGTAGACCTGCCCAGACTCG
IL-7-F(homo)	ACTTCCTCCCCTGATCCTTG	LTa-beta-F(homo)	CTGCTGCTCACCTCATTGGA
IL-7-R(homo)	GCTGACCATTAGAACACTCTCA	LTa-beta-R(homo)	AGACCACCTGGGAGTAGACG

Primers were designed and synthesized by Fuzhou Sunya Biotechnology Co.,LTD.

Supplementary Table 4. The catalogue numbers and dilutions and of antibodies.

Name	Catalogue number	Clone	Dilutions for usage
MLKL	Santa Cruz Biotechnology, Cat# sc-293201	3B2	1:1000 for immunoblotting<br>1:200 for immunostaining and immunohistochemistry
p-MLKL	ABclonal, Cat#AP0949	polyclones	1:1000 for immunoblotting<br>1:200 for immunostaining and immunohistochemistry
RIPK1	Abcam, Cat# ab178420<br>Cell Signal, Cat#73271	EPR4689-100<br>E8S7U	1:1000 for immunoblotting<br>1:200 for immunohistochemistry
p-RIPK1	Proteintech, Cat#28252-1-AP	polyclones	1:1000 for immunoblotting<br>1:200 for immunohistochemistry
RIPK3	Abcam, Cat# ab305054<br>Cell Signal, Cat#10188	EPR24374-135<br>E7A7F	1:1000 for immunoblotting<br>1:200 for immunohistochemistry
p-RIPK3	Abcam, Cat#ab209384	EPR9627	1:1000 for immunoblotting<br>1:200 for immunohistochemistry
Caspase 3	Cell Signal, Cat#9662	/	1:1000 for immunoblotting
Caspase 8	Abbkine, Cat#ABP0023	D384	1:1000 for immunoblotting
E-Cadherin	HUABIO, ET1607-75	SY0287	1:1000 for immunoblotting<br>1:200 for immunostaining
N-Cadherin	HUABIO, M1304-1	A10-4	1:1000 for immunoblotting
Vimentin	HUABIO, Cat#ET1610-39	SC60-05	1:1000 for immunoblotting<br>1:200 for immunohistochemistry
Snail	ABclonal, Cat#A5243	polyclones	1:1000 for immunoblotting
ZEB1	HUABIO, Cat#HA721438	PSH0-61	1:1000 for immunoblotting
MMP-2	Cell Signaling, Cat#40994	D4M2N	1:1000 for immunoblotting<br>1:200 for immunostaining
MMP-9	Cell Signaling, Cat#15749SF	D6O3H	1:1000 for immunoblotting<br>1:200 for immunostaining
MMP-12	HUABIO, Cat#ET1602-42	SR03-23	1:1000 for immunoblotting<br>1:200 for immunostaining
MPO	HUABIO, Cat#RT1410	2G3	1:1000 for immunoblotting
CitH3	Abcam, Cat#ab281584	RM1001	1:1000 for immunoblotting<br>1:200 for immunostaining

CXCL8	Cell Signaling, Cat#94407	E5F5Q	1:1000 for immunoblotting<br>1:200 for immunostaining
CD47	Abcam, Cat#ab218810	ab218810	1:1000 for immunoblotting<br>1:200 for immunostaining and immunohistochemistry
SIRPα	HUABIO, Cat#EM1902-37	A3E3	1:1000 for immunoblotting
CD24	Proteintech, Cat#18330-1-AP	polyclones	1:1000 for immunoblotting
SIGLEC10	HUABIO, Cat#EER1916-59	polyclones	1:1000 for immunoblotting
iNOS	Abcam, Cat#ab283655	RM1017	1:1000 for immunoblotting<br>1:200 for immunohistochemistry
Ki-67	Abcam, Cat#ab16667	SP6	1:1000 for immunoblotting<br>1:200 for immunohistochemistry
CXCR1	Abcam, Cat#ab124344	polyclones	1:1000 for immunoblotting
CXCR2	Abcam, Cat#ab65968	polyclones	1:1000 for immunoblotting
IL6R	Cell Signaling, Cat#18935S	E5B3P	1:1000 for immunoblotting
GM-CSFR	Cell Signaling, Cat#69817S	E8Z3S	1:1000 for immunoblotting
ICAM1	ABclonal, Cat#A22596	ARC53633_ARC54995	1:1000 for immunoblotting
PCNA	HUABIO, Cat#ET1605-38	SY12-07	1:1000 for immunoblotting
GAPDH	Abcam, Cat#ab181602	EPR16891	1:1000 for immunoblotting
F4/80	HUABIO, Cat#HA721745	PSH01-87	1:1000 for immunoblotting<br>1:200 for immunohistochemistry
CD34	HUABIO, Cat# ET1606-11	SI16-01	1:200 for immunohistochemistry
CD11b-PE	Proteintech, Cat#PE-65116	ICRF44	5 μL/test for flow cytometry
CD45-APC	eBioscience Invitrogen™, Cat#17-9459-42	2D1	5 μL/test for flow cytometry
CD80-FITC	eBioscience Invitrogen™, Cat#11-0809-42	B7-1	5 μL/test for flow cytometry
CD86-APC	eBioscience Invitrogen™, Cat#17-0869-42	B7-2	5 μL/test for flow cytometry
CD163-PE-Cyanine7	eBioscience Invitrogen™, Cat#25-1639-42	GHI/61	5 μL/test for flow cytometry
CD206-PE	eBioscience Invitrogen™, Cat#12-2069-42	19.2	5 μL/test for flow cytometry
Goat Anti-Rabbit IgG H&L HRP	HUABIO, Cat#HA1001	polyclones	1:50000 for immunoblotting
Goat Anti-Mouse IgG H&L HRP	HUABIO, Cat#HA1006	polyclones	1:50000 for immunoblotting
