# Supplementary material for: Necroptosis enhances ‘don’t eat me’ signal and induces macrophage extracellular traps to promote pancreatic cancer liver metastasis
Source: Nat Commun. 2024 Jul 18;15:6043. doi: 10.1038/s41467-024-50450-6 (PMC11258255; doi:10.1038/s41467-024-50450-6)
Supplement: Supplementary file 3 — Reporting Summary [file 41467_2024_50450_MOESM3_ESM.pdf]

nature portfolio

Corresponding author(s): Shi Chen

Last updated by author(s): Jun 22, 2024

Reporting Summary

Nature Portfolio wishes to improve the reproducibility of the work that we publish. This form provides structure for consistency and transparency in reporting. For further information on Nature Portfolio policies, see our Editorial Policies and the Editorial Policy Checklist.

Statistics

For all statistical analyses, confirm that the following items are present in the figure legend, table legend, main text, or Methods section.

n/a	Confirmed	
☐	☒	The exact sample size (*n*) for each experimental group/condition, given as a discrete number and unit of measurement
☐	☒	A statement on whether measurements were taken from distinct samples or whether the same sample was measured repeatedly
☐	☒	The statistical test(s) used AND whether they are one- or two-sided<br>*Only common tests should be described solely by name; describe more complex techniques in the Methods section.*
☒	☐	A description of all covariates tested
☒	☐	A description of any assumptions or corrections, such as tests of normality and adjustment for multiple comparisons
☐	☒	A full description of the statistical parameters including central tendency (e.g. means) or other basic estimates (e.g. regression coefficient) AND variation (e.g. standard deviation) or associated estimates of uncertainty (e.g. confidence intervals)
☐	☒	For null hypothesis testing, the test statistic (e.g. *F*, *t*, *r*) with confidence intervals, effect sizes, degrees of freedom and *P* value noted<br>*Give P values as exact values whenever suitable.*
☒	☐	For Bayesian analysis, information on the choice of priors and Markov chain Monte Carlo settings
☒	☐	For hierarchical and complex designs, identification of the appropriate level for tests and full reporting of outcomes
☒	☐	Estimates of effect sizes (e.g. Cohen's *d*, Pearson's *r*), indicating how they were calculated

Our web collection on statistics for biologists contains articles on many of the points above.

Software and code

Policy information about availability of computer code

Data collection	RT-qPCR data were acquired using StepOne™ Real-Time PCR Systems with StepOne™ software (version 2.2.3); Western blots were imaged by Bio-Rad ChemiDoc Imaging System using automatic exposure settings; Flow cytometry data were acquired with BD Accuri C6 Plus Flow Cytometer and the conected with BD Accuri C6 Plus Software (version 1.0.27.1).
Data analysis	Western Blot was detected by Enhanced Chemiluminescence using Smart-ECL Super kit (smart-lifesciences, S32500) and quantification was done using Image J (version 1.53a), and further analysis and calculations were done using Microsoft Excel and GraphPad Prism (version 8). Analysis of qPCR results were done using Microsoft excel and GraphPad Prism (version 8). All RNA-seq data was analyzed using R studio (https:///rstudio.com/)

For manuscripts utilizing custom algorithms or software that are central to the research but not yet described in published literature, software must be made available to editors and reviewers. We strongly encourage code deposition in a community repository (e.g. GitHub). See the Nature Portfolio guidelines for submitting code & software for further information.

Data

Policy information about availability of data

All manuscripts must include a data availability statement. This statement should provide the following information, where applicable:

- Accession codes, unique identifiers, or web links for publicly available datasets
- A description of any restrictions on data availability
- For clinical datasets or third party data, please ensure that the statement adheres to our policy

The related sequencing data in our study have been uploaded in the National Center for Biotechnology Information Sequence Read Archive (GSE248494). The other data generated in this study are available within the article, Source data and Supplementary Information. More information is available upon request from the corresponding author.

Research involving human participants, their data, or biological material

Policy information about studies with human participants or human data. See also policy information about sex, gender (identity/presentation), and sexual orientation and race, ethnicity and racism.

Reporting on sex and gender	This study did not involve sex or gender-based analysis. And sex was not considered in the study design.
Reporting on race, ethnicity, or other socially relevant groupings	This information has not been collected.
Population characteristics	Twelve patients who were pathologically diagnosed with T1 stage PDAC with or without liver metastasis were enrolled in this study. None of these patients received any previous therapy. According to the presence of liver metastasis, twelve patients were enrolled into T1M0 group (n=6) and T1M1 group (n=6) .
Recruitment	The informed consent was obtained from patients who received a craniotomy operation at Fujian Provincial Hospital. The surgical resection or biopsy specimens were then subjected to experiments. There is no potential self-selection bias.
Ethics oversight	Ethical approval for this study was obtained from the Ethics Committee of Fujian Provincial Hospital.

Note that full information on the approval of the study protocol must also be provided in the manuscript.

Field-specific reporting

Please select the one below that is the best fit for your research. If you are not sure, read the appropriate sections before making your selection.

☒ Life sciences ☐ Behavioural & social sciences ☐ Ecological, evolutionary & environmental sciences

For a reference copy of the document with all sections, see nature.com/documents/nr-reporting-summary-flat.pdf

Life sciences study design

All studies must disclose on these points even when the disclosure is negative.

Sample size	For in vitro experiments, at least triplicates were included to enable statistical calculations. For in vivo studies, a sample size of 3-8 animals per treatment group was deemed sufficient to reliably detect statistically significant differences.
Data exclusions	No data were excluded from the analyses.
Replication	The experiments were replicated, and the findings were reproducible. Findings were replicated in at least three biological independent samples each.
Randomization	Cells or mice were randomly assigned to different groups before treatment.
Blinding	The investigators maintained awareness of the experimental conditions throughout the data acquisition and analysis process, and the investigators were unbiased in both outcome assessment and conclusion drawing.

Reporting for specific materials, systems and methods

We require information from authors about some types of materials, experimental systems and methods used in many studies. Here, indicate whether each material, system or method listed is relevant to your study. If you are not sure if a list item applies to your research, read the appropriate section before selecting a response.

Materials & experimental systems

n/a	Involved in the study
☐	☒ Antibodies
☐	☒ Eukaryotic cell lines
☒	☐ Palaeontology and archaeology
☐	☒ Animals and other organisms
☒	☐ Clinical data
☒	☐ Dual use research of concern
☒	☐ Plants

Methods

n/a	Involved in the study
☒	☐ ChIP-seq
☐	☒ Flow cytometry
☒	☐ MRI-based neuroimaging

Antibodies

Antibodies used

MLKL (Santa Cruz Biotechnology, Cat# sc-293201)
p-MLKL (ABclonal, Cat#AP0949)
RIPK1 (Abcam, Cat# ab178420; Cell Signal, Cat#73271)
p-RIPK1 (Proteintech, Cat#28252-1-AP)
RIPK3 (Abcam, Cat# ab305054; Cell Signal, Cat#10188)
p-RIPK3(Abcam, Cat#ab209384)
Caspase 3 (Cell Signal, Cat#9662)
Caspase 8 (Abbkine, Cat#ABP0023)
E-Cadherin (HUABIO, ET1607-75)
N-Cadherin (HUABIO, M1304-1)
Vimentin (HUABIO, Cat#ET1610-39)
Snail (ABclonal, Cat#A5243)
ZEB1 (HUABIO, Cat#HA721438)
MMP-2 (Cell Signaling, Cat#40994)
MMP-9 (Cell Signaling, Cat#15749SF)
MMP-12 (HUABIO, Cat#ET1602-42)
MPO (HUABIO, Cat#RT1410)
CitH3 (Abcam, Cat#ab281584)
CXCL8 (Cell Signaling, Cat#94407)
CD47 (Abcam, Cat#ab218810)
SIRPα (HUABIO, Cat#EM1902-37)
CD24(Proteintech, Cat#18330-1-AP)
SIGLEC10(HUABIO, Cat#EER1916-59)
iNOS (Abcam, Cat#ab283655)
Ki-67 (Abcam, Cat#ab16667)
CXCR1 (Abcam, Cat#ab124344)
CXCR2 (Abcam, Cat#ab65968)
IL6R (Cell Signaling, Cat#18935S)
GM-CSFR (Cell Signaling, Cat#69817S)
ICAM1 (ABclonal, Cat#A22596)
PCNA (HUABIO, Cat#ET1605-38)
GAPDH (Abcam, Cat#ab181602)
F4/80(HUABIO, Cat#HA721745)
CD34(HUABIO, Cat#ET1606-11)
CD11b (PE, Proteintech, Cat#PE-65116)
CD45 (APC, eBioscience Invitrogen™, Cat#17-9459-42)
CD80 (FITC, eBioscience Invitrogen™, Cat#11-0809-42)
CD86 (APC, eBioscience Invitrogen™, Cat#17-0869-42)
CD163 (PE-Cyanine7, eBioscience Invitrogen™,Cat#25-1639-42)
CD206 (PE, eBioscience Invitrogen™, Cat#12-2069-42)
Goat Anti-Rabbit IgG H&L (HRP) (HUABIO, Cat#HA1001)
Goat Anti-Mouse IgG H&L (HRP) (HUABIO, Cat#HA1006)
For immunoblotting, antibodies were diluted as 1:1000. For immunostaining and immunohistochemistry, antibodies were diluted as 1:200. For flow cytometry, antibodies were added 5 μL (0.25 μg) per test.

Validation

All antibodies used in this study were validated by manufacturers for that specific application. Relevant validating results can be found in the website of each manufacturer.

Eukaryotic cell lines

Policy information about cell lines and Sex and Gender in Research

Cell line source(s)

Human PDAC cells (PANC-1, CRL-1469; AsPC-1, CRL-1682) and myeloid cell lines THP1 (TIB-202) and RAW 264.7 (TIB-71) were obtained from American Type Culture Collection (ATCC, Manassas, VA, USA). HUVEC cells (CL-0675) were purchased from Pricella Life Science&Technology Co.,Ltd.

Authentication	All cell lines used in this study were authenticated by the supplier. Cell authentication is based on their morphology, growth conditions and specific gene expression.
Mycoplasma contamination	All cell lines were tested negative for mycoplasma contamination.
Commonly misidentified lines (See ICLAC register)	No commonly misidentified lines were used in this study.

Animals and other research organisms

Policy information about studies involving animals; ARRIVE guidelines recommended for reporting animal research, and Sex and Gender in Research

Laboratory animals	Six to eight weeks old male and female C57BL/6 and NOD-SCID mice were used in this study. All mice were purchased from the Anburui BD Laboratory. KPC mice were obtained from Shanghai Model Organisms Center. All mice used in this study were age-littermates. All mice used in this study were age- and sex-matched littermates. Both male and female mice were used in this study.
Wild animals	The study did not involve wild animals.
Reporting on sex	The study did not involve sex-based analysis.
Field-collected samples	The study did not involve filed-collected samples.
Ethics oversight	All animals were handled strictly according to the Principles for the Utilization and Care of Vertebrate Animals and the Guide for the Care and Use of Laboratory Animals. All animal experiments were approved by the Institutional Animal Care and Use Committee of Fujian Medical University.

Note that full information on the approval of the study protocol must also be provided in the manuscript.

Plants

Seed stocks	N/A
Novel plant genotypes	N/A
Authentication	N/A

Flow Cytometry

Plots

Confirm that:

☒ The axis labels state the marker and fluorochrome used (e.g. CD4-FITC).

☒ The axis scales are clearly visible. Include numbers along axes only for bottom left plot of group (a 'group' is an analysis of identical markers).

☒ All plots are contour plots with outliers or pseudocolor plots.

☒ A numerical value for number of cells or percentage (with statistics) is provided.

Methodology

Sample preparation	For macrophage polarization assays, Harvested single cell suspensions of THP-1 derived macrophages were washed with Flow Cytometry Staining Buffer (Invitrogen™), and then treated with an FcR-blocker (BD Pharmingen™) for 10 min at 4 °C. Then, different antibodies from Thermo-Fisher (FITC-CD80, APC-CD86, PeCy7-CD163,PE-CD206, 5ul each sample) were added to incubate for 20min at 25°C. After staining was completed, the cells were gently washed three times with Flow Cytometry Staining Buffer (Invitrogen™) for further analysis.<br>For flow cytometry-based in vitro phagocytosis assays, tumor cells expressed virally GFP and macrophages were co-cultured in ultra-low-attachment 96-well U-bottom plates (Corning, 4515) in serum-free RPMI (Thermo Fisher Scientific). Plates were washed two times; macrophages were added to the plate; and plates were then incubated for 2h at 37°C. Phagocytosis was analyzed by flow cytometry, and measured as the number of APC-CD45+(17-9459-42, Thermo-Fisher), PE-CD11b+(65116, Proteintech) and GFP+ macrophages as a percentage of the total CD11b+ macrophages. For further treatment, IgG or Anti-CD47 at a concentration of 10μg/mL were incubated with tumor cells for 20 min hours in a humidified 5% CO2 incubator at 37°C before co-incubated with macrophage.

Instrument	BD Accuri C6 Plus Flow Cytometer
Software	BD Accuri C6 Plus Software (version 1.0.27.1) and FlowJo software (version 10.8.1)
Cell population abundance	Cell population abundances were analyzed using FlowJo software. This study did not involve cell-sorting experiments.
Gating strategy	Flow cytometry gating strategy for THP-1-derived macrophage (CD45+ CD11b+) tumor phagocytosis events (GFP+). Cells were selected by size using SSC-A vs FSC-A; CD11b was gated to separate macrophages, then FITC were gated to detect GFP-labeled tumor cells uptake of macrophages.

☒ Tick this box to confirm that a figure exemplifying the gating strategy is provided in the Supplementary Information.
